# Epidemiologic Determinants Affecting Cigarette Smoking Cessation: A Retrospective Study in a National Health System (SSN) Treatment Service in Rome (Italy)

**DOI:** 10.1155/2010/183206

**Published:** 2010-04-13

**Authors:** Maria Giulia Marino, Elisabetta Fusconi, Rosanna Magnatta, Augusto Panà, Massimo Maurici

**Affiliations:** ^1^Department of Public Health, University of Roma Tor Vergata, via Montpellier 1, 00133 Roma, Italy; ^2^Smoking Treatment Service, Local Health District 9 Roma C via Monza 2, 00182 Roma, Italy

## Abstract

This retrospective study aims to evaluate epidemiologic characteristics of patients attending stop smoking courses, based on group therapy, testing their influence on smoking cessation in univariate and multivariate model. A total of 123 patients were included in this study. Mean age was 53 (±11). Sixty-seven percent were women. At the end of the courses 66% of patients stopped smoking, after 12 months only 39% remained abstinent. Patients younger than 50 years statistically tended to continue smoking 6 months (*P* = .02–R.R. = 1.49, C.I. 95%: 1.06–2.44) and 12 months (*P* = .03–R.R. = 1.37, C.I. 95%: 1.02–2.52) after the end of the courses. A low self-confidence in quitting smoking was significantly related to continuing tobacco consumption after 6 months (*P* = .016–R.R. = 1.84, C.I. 95%: 1.14–2.99). Low adherence to therapeutic program was statistically associated to maintenance of tobacco use at 6 months (*P* = .006–R.R. = 1.76, C.I. 95%: 1.32–2.35) and 12 months (*P* = .050–R.R. = 1.45, C.I. 95%: 1.11–1.88). This association was confirmed at 6 months in the analysis performed on logistic regression model (*P* = .013).

## 1. Introduction

Italy has recently ratified the Framework Convention on Tobacco Control (FCTC), approved by World Health Assembly (WHA) in 2003 [1], and was among first countries to promote a “smoke free” society implementing effective strategies for smoking prevention [2].

In Italy, as in all developed countries, cigarette smoking is the main avoidable cause of morbidity and mortality [3]. Worldwide there are over a billion smokers [4], with Italy having 11.2 million smokers (about 22% of the population over age 15). In Italy, the number of smokers shows a decreasing trend in recent years; in 2008 the reduction in prevalence was 1.5% from 2007. About 90% are daily smokers, more than half smoke >15 cigarettes [5].

Male Italian smokers belong mainly to the 25–44 age group, while females are in the 45–64 range; young smokers (<24 years) number about 1.5 million [5]. These characteristics are different from what is observed in smoking treatments services in which patients are usually elderly [3]. 

A direct correlation between smoking (including second-hand smoke) and neoplastic, respiratory and cardiovascular diseases is widely demonstrated [6–8]. Tobacco use during pregnancy is a well-known risk factor for low birth weight or abortion; exposure to second hand smoke is related to an increased risk of sudden infant death syndrome, otitis media, respiratory tract infections and asthma in children [9]. Smoking-induced deaths in Italy reach nearly 85 000 each year (25% in the age group 35–65) [10], and about 15% of deaths are smoking related [11]. 

Several studies have demonstrated that quitting smoking is related to a reduction of risk of illness and to an increased life span, especially in young people and also evident in the elderly [12]. 

About one-third of Italian smokers have attempted to quit smoking [5]. Smoking cessation measures have shown partial adherence in Italy and one of the main goals of the Local Health Prevention Services is to reach maximum percentage of long lasting abstinent patients in quit smoking treatments. Therapeutic approaches are varied and evolving, therefore it is essential to enhance awareness about the factors affecting the success of such treatments. This would enable to personalize therapies and, consequently, give a greater number of smokers the possibility of long-lasting cessation. 

This study aims to

- evaluate epidemiologic factors of patients attending stop smoking courses in a National Health System (SSN) treatment service in Rome, identifying determinants that influence cessation of cigarette smoking;
- propose a suitable methodology for Public Health personnel to help them to improve treatment success rating.

## 2. Materials and Methods

**Figure 1:**
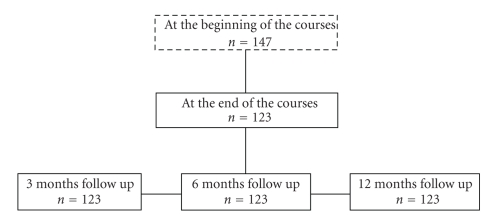
Study flow chart.

The retrospective study (Figure 1) has included patients frequenting seven stop smoking courses organized from 2003 to 2005 by an SSN treatment service in the centre of Rome, whose attendance was free of charge and accessible to all inhabitants who decided to participate in.

The stop smoking courses were based on a group therapy called *Gruppo di Fumatori in Trattamento* –Treated Smokers Group, inspired to the Five-Day Plan of McFarland et al. [13]. This program was based on a cognitive-behavioural approach consisting of five steps (preparedness, full immersion, maintenance, involvement, and further aid) for a total of 10 meetings; smokers were forced to stop abruptly (cold turkey method) at the third day of the course [14].

We included in the analysis all patients who did not withdraw before third day of the courses; follow-up of patients was conducted directly or through the phone at the end of the courses and 3, 6, and 12 months later to verify smoking abstinence. We decided to consider “lost to follow-up” subjects unreachable by phone after 3 attempts in different days. 

During former interviews socio-demographic characteristics (gender, age, occupation, marital status, and education level), information on habits and smoke addiction (sports activity, coffee use, alcohol consumption; age of first use of cigarettes, number of cigarettes smoked per day, years of addiction, quit smoking attempts and principal reason to stop smoking), some clinical features (weight, height, blood pressure and heart rate) and other data (living with other smokers or having smokers in the family, information sources about treatment services, illnesses and/or risk factors) were collected. Furthermore two tests were administered to patients: Fagerstrom Test to evaluate nicotine dependence [15] and Self-efficacy Test to estimate the belief in one's own capability to stop smoking measured on a scale of 1 to 10. The Fagerstrom Test based on a scale of 0 to 10 is directly related to dependence severity: 0–2 low, 3-4 medium, 5-6 high, 7–10 very high.

All anamnestic and follow-up data were collected in a Microsoft Excel database. A unique identifying alphanumeric code was assigned to every patient to preserve their privacy. Quit rates were evaluated 6 and 12 months after the end of the courses. 

We considered “early” smokers patients who started smoking before the age of 15 and “long lasting” those who had smoked for more than 20 years. 

We classified as “heavy” smokers patients who smoked ≥20 cigarettes/day.

Education was divided into “higher” (academic degree and high school) and “lower” (primary and secondary school) levels. Body Mass Index (BMI: weight/height^2^) was measured and patients were stratified in four groups: BMI < 20, 20 ≤ BMI<25, 25 ≤ BMI < 30, and BMI ≥ 30.

The working group decided to consider “confident” in their capabilities to stop smoking subject with scores ≥7 in Self-Efficacy Test and “highly nicotine-addicted” those with score ≥5 in Fagerstrom Test.

Methods that have not been explicitly defined were freely chosen by the working group.

EPI-INFO 3.3 Software (trademark of the Centers for Disease Control and Prevention (CDC)) was used for statistical analysis. Student's *t*-test for unpaired data was used to test statistically significant differences between heart rate averages measured at the beginning and end of the courses and differences in age between the sexes. 

The influence of epidemiologic characteristics on smoking cessation in univariate and multivariate analysis was tested, calculating Relative Risks (RR) with confidence intervals of 95% (C.I. 95%). 

Statistical threshold was set at.05 and evaluated with chi-square test (with Yates' correction when needed). We chose to enter into logistic regression model variables resulting in *P* < .15 in univariate analysis. Good fit of the model was checked by EPI-INFO likelihood ratio.

## 3. Results

From a total of 147 subjects who have started frequenting stop smoking courses, 123 (83.7%) attended all course meetings. No one was lost to follow-up (Figure 1). Age range was between 29 and 76 years, and mean age was 53 (±11) without statistical differences between the sexes. Sixty-seven percent were women (52% of them in menopause). Socio-demographic and epidemiological characteristics are illustrated in Table 1, while smoking habits are shown in Table 2.

The average of courses frequency and self-efficacy test were 8/10 (±1.8) and 7.4/10 (±2.4), respectively; 64.4% of patients declared themselves “confident” in quitting smoking. 

At the end of the courses, 81 patients (66%) stopped smoking; patients who did not abstain lowered their tobacco consumption about 40% (±37.2%). Data about heart rate, measured at the beginning and end of the courses in 53 patients, showed a statistical significant mean reduction of 16 beats, tested with Student's *t*-test (*P* < .0001). 

Three months after the end of courses, 58% of patients were still abstinent; after 6 and 12 months, this value decreased to 47% and 39%, respectively.

Patients younger than 50 years statistically tended to continue smoking 6 months (*P* = .02–R.R. = 1.49, C.I. 95%: 1.06–2.44) and 12 months (*P* = .03–R.R. = 1.37, C.I. 95%: 1.02–2.52) after the end of the courses.

A low self-confidence in the possibility of cessation of smoking (self-efficacy test <7) was significantly related to continuing tobacco consumption after 6 months (*P* = .016– R.R. = 1.84, C.I. 95%: 1.14–2.99); this relation was not significant when tested after 12 months (*P* = .058). 

Low adherence to therapeutic program, defined as having attended less than 7/10 meetings, was statistically associated to maintenance of tobacco use at 6 months (*P* = .006–R.R. = 1.76, C.I. 95%: 1.32–2.35) and 12 months (*P* = .050– R.R. = 1.45, C.I. 95%: 1.11–1.88). 

Failure in therapy was related—although not significantly—to being a “heavy” smoker (at 6 months *P* = .07– R.R. = 1.45, C.I. 95%: 0.93–2.26), having a smoking father (at 12 months *P* = .08–R.R. = 1.61, C.I. 95%: 0.87–2.97), and being “highly nicotine-addicted” (at 6 months *P* = .12– R.R. = 1.34, C.I. 95%: 0.90–1.98).

Analysis performed on logistic regression model confirmed the association between continuing smoking after 6 months and low frequency of courses (*P* = .013) (Likelihood ratio 16,78–*P* = .01). For low self-confidence, a relationship close to the upper limit for significance (*P* = .052) was observed; no association was confirmed at 12 months (Table 3).

An intermediate multivariate analysis performed to verify if course frequency effect could have had some correlations with other baseline information (Age and Self efficacy) showed no significant association.

The influence of several variables on smoking cessation such as gender, occupation, age of first use of cigarettes, years of addiction, marital status, BMI, living with other smokers, education level, quit smoking attempts, sports activity, coffee use, principal reason to stop smoking, information sources about treatment services, illness and/or presence of risk factors and alcohol consumption were tested; these data showed no significant association.

## 4. Discussion

Although several studies confirm that cessation of smoking is possible even without any kind of therapeutical approach [16, 17], most authors have highlighted that the best rate of success in smoking cessation can be obtained through pharmacological and psychological therapies, especially when combined [3, 18, 19]. Remarkably, the higher percentage of abstinent patients 6 months after the end of the therapy seems attainable by administering nicotine replacement therapy together with group therapy [3, 20]. Nevertheless these evidences, there is still a large number of patients receiving stop smoking therapies that are not able to free themselves from tobacco dependence.

For this reason in the last years factors influencing smoking cessation became the focus of many studies in order to improve success of such therapies.

Our treatment service is the only National Health System smoke cessation centre of a metropolitan area that counts about 127.324 inhabitants [21]. Although this cohort is not representative of Italian population, it shows the same general features of all patients attending smoking cessation services [3].

Unlike some recent reports [3, 5], the majority of people in our sample are women; this peculiarity could be explained by the female preference for group therapy than other types of stop smoking approaches found also in other studies [20, 22, 23]. Mean age of patients attending these stop smoking courses is higher than that reported by Italian and global smokers statistics [3, 20, 24]; this could be related to the demographic profile of the area in which the service is located, which presents an high percentage of elder (over 65 years old) inhabitants [21].

Regarding education level, official data show that the majority of Italian smokers have a medium to low education level [17]; however patients attending Italian stop smoking services usually have a higher one, as highlighted in our study [3, 24].

In this cohort, the percentage of “heavy” smokers is almost twice than the general Italian smoker population [25], but, however, overlaps other articles [3, 20]; the same ratio holds good with regard to the percentage of “long lasting” smokers (in this cohort about 90%). Degree of nicotine dependence shown in this study is similar to the reports in literature [3, 20].

Regarding patients who previously attempted to quit smoking, the evidences obtained from this study demonstrate a higher percentage versus the national data (67% versus 30%); this corresponds to the results shown in an Italian multicentric study [3]. In our sample the principal reason to attempt to stop smoking is related to health matters as reported by other authors [26].

Considering percentage of abstinent patients, the results of the treatment of this study are higher than reported by other group therapy-based studies, both at 6 months [3, 20] and 12 [20, 24] months after the end of courses.

The lack of an objective measure to verify patients' smoking cessation could represent a limitation; however, in almost all the SSN smoking cessation services the standard procedure is to follow up patients by phone. On the other hand it is important to stress the psychological approach of group therapy, that makes the smoker responsible for successful cessation and the person in charge of follow-up more confident of truthfulness of patient's answers. 

This study highlights a statistically significant risk of continuing smoking in subjects younger than 50 years. This is confirmed by several authors [27–29], but not found by others [30]; a reasonable explanation could be the low importance that younger people give their health status. The findings in literature, confirmed by the results obtained in this study, relate significantly low self-confidence in the possibility to stop smoking [31–33] and low adherence to therapeutic program [3, 24, 34] with failure in therapy. 

In logistic analysis only the relation between failure in smoking treatment success and low attending frequency (<7) was confirmed; we would like to highlight that relation with low self confidence reached a significance close to the upper limits, making us still considering it a deserving attention factor.

The differences observed in univariate and multivariate analysis results suggested us to perform an intermediate multivariate analysis in order to distinguish the importance of tested variables and to evaluate possible influence of age and self efficacy on course frequency effect. The results of this analysis showed us a uniform effect of course frequency with respect to baseline information considered, confirming us the reliability of main results of our study. 

Self-reported cigarette consumption per day [35–37] and degree of nicotine dependence [38–40] do not show statistical correlations with the capability to stop smoking as found in other studies. Likewise, several articles have shown a direct relation between smoking cessation and gender [41–43], first cigarette use in post teenage years [41, 42, 44], years of addiction [20], high BMI [34, 45, 46], living with nonsmoker flatmates or having a smoking parent [47–49], education level [28, 50, 51], previous attempt to stop smoking [42], marital status [3, 28, 52] and occupation [37, 45].

In our Country there are few reliable data about the relation between alcohol and tobacco consumption. In our sample this well-known association [48, 53] is not confirmed, as reported in Table 1. This founding is probably related to the setting in which the study was conducted (in an area of the centre of Italy): in fact although we observed in the last years an increase of alcohol consumption in general Italian population, there are still geographical differences between northern and centre/southern regions in which we still found a lighter alcohol consumption [54].

In the light of our results there are some suggestions to better target stop smoking treatment services interventions: for instance, low self-confidence in the possibility to stop smoking and the low adherence to a therapeutic program could be improved enhancing psychological motivation. For younger patients could be useful adding pharmacological therapy to the psychological one, obtaining a well known successful approach. Furthermore, information, education and communication campaigns, whose effectiveness was recently demonstrated in a study pointed on radio commercials and internet advertisements [55], could be focused on population of <50 years old.

## 5. Conclusion

Results obtained from this study highlight some factors, which should be considered when planning therapeutic approaches for smoking cessation. 

In the future, all smoking treatment services should investigate epidemiologic determinants affecting the cessation of cigarette smoking in patients attending their therapeutic programs to improve the effectiveness of interventions and implement the most suitable approach for the target population.

## Figures and Tables

**Table 1:** Sociodemographic and epidemiological features of population in study.

Sociodemographic and epidemiological features of population in study	Smokers^§^ (*n* = 65)		Abstinents^§^ (*n* = 58)		Total (*n* = 123)	
	*n*	%	*n*	%	*n*	%
Gender						

male	18	43.9%	23	56.1%	41	33.3%
female	47	57.3%	35	42.7%	82	66.7%

Age groups						

≤40	11	57.9%	8	42.1%	19	15.4%
41–50	25	67.6%	12	32.4%	37	30.1%
51–60	13	41.9%	18	58.1%	31	25.2%
>60	16	44.4%	20	55.6%	36	29.3%
mean (SD)	51.5 (11)		54.3 (11.2)		52.8 (11.2)	∗
median (min-max)	48 (33–76)		54.5 (29–76)		52 (29–76)	∗

Information sources about treatment service						

friends/relatives	23	54.8%	19	45.2%	42	34.1%
brochures edited by local health service	13	46.4%	15	53.6%	28	22.8%
medical staff	7	43.8%	9	56.3%	16	13.0%
other	15	68.2%	7	31.8%	22	17.9%
missing information	6	40.0%	9	60.0%	15	12.2%

Occupation						

employee	32	58.2%	23	41.8%	55	44.7%
freelancer	6	60.0%	4	40.0%	10	8.1%
retired	14	43.8%	18	56.3%	32	26.0%
other	13	50.0%	13	50.0%	26	21.1%

Education level						

primary	1	50.0%	1	50.0%	2	1.6%
secondary	12	46.2%	14	53.8%	26	21.1%
high	35	53.8%	30	46.2%	65	52.8%
degree	17	56.7%	13	43.3%	30	24.4%

Marital status						

single	20	66.7%	10	33.3%	30	24.4%
married/live-in partner	25	43.9%	32	56.1%	57	46.3%
legally separated/divorced	17	68.0%	8	32.0%	25	20.3%
widow	3	27.3%	8	72.7%	11	8.9%

BMI						

<20	5	62.5%	3	37.5%	8	6.5%
≥20 e <25	30	52.6%	27	47.4%	57	46.3%
≥25 e <30	26	56.5%	20	43.5%	46	37.4%
≥30	3	42.9%	4	57.1%	7	5.7%
missing information	1	20.0%	4	80.0%	5	4.1%
mean (SD)	24.0 (3.3)		24.7 (3,3)		24.3 (3.3)	
median (min-max)	24.1 (18.4–32.1)		24.7 (17.7–34)		24.2 (17.7–34)	

Sports activity						

no	35	56.5%	27	43.5%	62	50.4%
rarely	8	57.1%	6	42.9%	14	11.4%
daily	13	50.0%	13	50.0%	26	21.1%
missing information	9	42.9%	12	57.1%	21	17.1%

Illness and/or risk factors (more factors for each patient)						

cardiovascular	37	56.9%	28	43.1%	65	∗
respiratory	44	61.1%	28	38.9%	72	∗
gastric	21	56.8%	16	43.2%	37	∗
psychiatric	8	100.0%	0	0.0%	8	∗
other	15	50.0%	15	50.0%	30	∗
missing information	1	9.1%	10	90.9%	11	∗

Principal reason to stop smoking (more factors for each patient)						

Health	22	32.4%	46	67.6%	68	∗
External pressure	8	32.0%	17	68.0%	25	∗
Self-esteem	10	41.7%	14	58.3%	24	∗
Economic	7	46.7%	8	53.3%	15	∗
Other	9	30.0%	21	70.0%	30	∗

Coffee use						

≤2	21	65.6%	11	34.4%	32	26.0%
>2 e ≤4	24	46.2%	28	53.8%	52	42.3%
>4	20	51.3%	19	48.7%	39	31.7%

Alcohol consuption						

yes	23	53.5%	20	46.5%	43	35.0%
no	42	52.5%	38	47.5%	80	65.0%

^§^at 6 months after the end of the course.

**Table 2:** Smoking habits of population in study.

Smoking habits of population in study	Smokers^§^ (*n* = 65)		Abstinents^§^ (*n* = 58)		Total (*n* = 123)	
	*n*	%	*n*	%	*n*	%
Cigarettes per day						

heavy smoker (≥20)	51	58.0%	37	42.0%	88	71.5%
light smoker (<20)	14	40.0%	21	60.0%	35	28.5%
mean (SD)	25.8 (11.5)		22.4 (11.1)		24.2 (11.4)	∗

Age of first use of cigarettes						

early smoker (<15aa.)	9	56.3%	7	43.8%	16	13.0%
late smoker (≥15aa.)	56	52.3%	51	47.7%	107	87.0%
mean (SD)	18.8 (4.8)		19.2 (5.1)		19.0 (4.9)	∗

Years of addiction						

<20	7	53.8%	6	46.2%	13	10.6%
≥20	58	52.7%	52	47.3%	110	89.4%
mean (SD)	31.6 (9.7)		33.2 (11.0)		32.3 (10.3)	∗

Quit smoking attempts						

none	24	60.0%	16	40.0%	40	32.5%
one or more	41	49.4%	42	50.6%	83	67.5%
*by oneself *	22	38.6%	35	61.4%	57	∗
*group therapy *	8	61.5%	5	38.5%	13	∗
*other *	11	52.4%	10	47.6%	21	∗
mean abstinence in months (SD)	9.2 (12.7)		20.3 (33.9)		16.6 (30.2)	∗

Fagerstrom test						

0–2 (low)	4	33.3%	8	66.7%	12	9.8%
3-4 (medium)	14	48.3%	15	51.7%	29	23.6%
5-6 (high)	22	52.4%	20	47.6%	42	34.1%
7–10 (very high)	25	65.8%	13	34.2%	38	30.9%
mean (SD)	5.9 (2.2)		4.9 (2,3)		5.4 (2,3)	∗

Living with other smokers						

no	40	57.1%	30	42.9%	70	56.9%
yes	25	47.2%	28	52.8%	53	43.1%

Smokers in the family						

no	2	22.2%	7	77.8%	9	∗
smoking father	29	49.2%	30	50.8%	59	∗
others	27	54.0%	23	46.0%	50	∗

^§^at 6 months after the end of the course.

**Table 3:** Univariate and multivariate analysis.

*Tested Variables*	6 months			6 months adjusted			12 months			12 months adjusted		
	R.R.	C.I. 95%	*P*-value	O.R. adj.	C.I. 95%	*P*-value	R.R.	C.I. 95%	*P*-value	O.R. adj.	C.I. 95%	*P*-value
Age												
>50 years	1			1			1			1		
≤50 years	1.49	1.06–2.44	.02	1.50	0.50–4.45	.46	1.37	1.02–2.52	.03	1.36	0.43–4.27	.6

Self-efficacy test												
≥7	1			1			1			1		
<7	1.84	1.14–2.99	.016	2.99	0.99–9.04	.052	1.47	1.01–2.14	.058	2.72	0.77–9.54	.12

Course frequency												
≥7	1			1			1			1		
<7	1.76	1.32–2.35	.006	8.70	1.56–48.36	.013	1.45	1.11–1.88	.05	3.41	0.6–19.29	.16

Cigarettes consumption												
<20/day	1			1								
≥20/day	1.45	0.93–2.26	.07	0.97	0.21–4.33	.97						

Nicotine dependence												
low degree (Fagestrom <5)	1			1								
high/very high degree (Fagestrom ≥5)	1.34	0.90–1.98	.12	0.81	0.21–3.22	.77						

Marital status												
married/live-in partner	1			1								
living alone	1.38	0.97–1.97	.06	2.04	0.71–5.88	.18						

Smokers in the family												
not smoking father							1			1		
smoking father							1.61	0.87–2.97	.08	2.48	0.74–8.26	.14

	Test		Statistic	D.F.	*P*-value		Test		Statistic	D.F.	*P*-value	
	Score		15.173	6	.019		Score		7.1913	4	.1261	
	Likelihood Ratio		16.7813	6	.0101		Likelihood Ratio		7.8354	4	.0978	
